# Transcriptome and Metabolome Studies on Pre-Harvest Nitrogen Impact on Fruit Yield and Quality of Peach (*Prunus persica* L.)

**DOI:** 10.3390/metabo12100905

**Published:** 2022-09-26

**Authors:** Yu Zhang, Jiying Guo, Xin Zhou, Jianbo Zhao, Xin Liu, Quan Jiang, Fei Ren

**Affiliations:** 1 Institute of Forestry and Pomology, Beijing Academy of Agriculture and Forestry Sciences, Beijing 100093, China; 2 Key Laboratory of Biology and Genetic Improvement of Horticultural Crops (North China), Ministry of Agriculture and Rural Affairs, Beijing 100093, China; 3 Beijing Engineering Research Center for Deciduous Fruit Trees, Beijing 100093, China; 4 Department of Pomology, College of Horticulture, China Agriculture University, Beijing 100193, China

**Keywords:** peach, different nitrogen levels, different growth stages, transcriptome analysis, metabolome analysis

## Abstract

Pre-harvest nitrogen (N) application has been proven effective for improving fruit yield and quality. However, a full understanding of how differences in N availability/plant N status influence the transcriptome and metabolism underlying yield formation and quality remains elusive. Here, a combined analysis of the morpho-physiological qualities, transcriptome, and metabolite of peach plants was performed under different nitrogen levels at fruit pit hardening (PH) and fruit expansion (FE). Nitrogen fertilizer directly affected the yield, fruit quality, and metabolites of peach at different growth stages. RNA-Seq was used to analyze the influence of N levels at PH and FE in peach. Kyoto Encyclopedia of Genes and Genomes (KEGG) pathway analysis showed that the differentially expressed genes (DEGs) focused on flavonoid biosynthesis and secondary metabolite biosynthetic processes. The differential metabolites among the different treatments were mainly involved in flavonoid metabolism. Transcriptome analysis showed that applying different nitrogen fertilizers at different growth stages of peach mainly affected the synthesis of flavonoids in fruit. Overall, these results suggest that the impacts of pre-harvest N application on fruit yield and quality differ between developmental stages. This research provides a full understanding of the metabolic processes underlying fruit growth and development in peach, providing a theoretical basis for the improvement of nitrogen use efficiency in peach trees.

## 1. Introduction

Nitrogen (N) is macro-essential for plant growth and development, determining crop yields through complex processes of N metabolism [1]. Due to the great contribution of N to crop production, farmers apply N fertilizer at the recommended dose for N fertilizers in order to achieve high harvest outcomes [2,3,4]. In China, the N utilization efficiency of crops is only 20–30%; far lower in comparison to other parts of the globe [5]. To date, plant response to low N availability has been well studied at the whole-plant, cellular, and molecular levels [6,7]. Recently, an integrative morpho-physiological, transcriptome, and metabolome analysis emerged, revealing the impacts of N sufficiency and deficiency conditions in plants of the rosaceae family, and guiding its appropriate fertilization practices [6]. Such combined transcriptomic and metabolomic or phenotypic analyses have also been studied in the carbon (C) and N interactions in rice under low-N stress, and root response to N and hormones in *Arabidopsis thaliana* [8,9]. Therefore, it is interesting to know how peach plant responds to N availability.

Perennial fruit trees have contrasting responses to low N availability during growth and development due to N assimilation, storage, and reallocation compared to annual herbaceous crops. Previous studies have found that increasing N supply from the blooming stage to three weeks before maturity resulted in improved leaf N status and photosynthetic activity, generating faster cell proliferation and larger ‘Gala’ apple fruit [10]. However, another study found that low doses of urea had no effect on flesh firmness, total soluble solids, or starch content during the cell enlargement stage in apple plants [11]. In mango, a higher proportion of N applied 2 or 4 weeks prior to harvest was able to increase the growth of leaves and stem, while low rates of N applied prior to harvest increased stem biomass only [12]. Previous results indicated that fruit crops have different responses to pre-harvest N application.

Peach (*Prunus persica* (L) Batsch), with a production of nearly 20 million tonnes of fruit per year, is one of the most economically important fruit crops among the Rosaceae family [13]. The development process of peach can generally be described as consisting of four recognized distinct growth stages (S1–S4) [14,15,16,17]. The first stage (S1), the first exponential growth phase, is characterized by a rapid increase in cell division and elongation. During the second stage (S2), the endocarp hardens to form the stone (pit hardening), and there is hardly any increase in fruit size, which is the pit hardening stage (PH) in our study [18]. During the third stage (S3), known as the second exponential growth phase, there is a rapid increase in fruit size and rapid cell division, corresponding to the fruit expansion stage (FE). In the final stage (S4), the fruit reaches its final full size and enters the fruit ripening or climacteric stage [17]. The sensitivity to N fertilizer was different in the four stages of fruit development. At present, the nitrite transporters (NRT) and ammonium transporter (AMT) participate in nitrate uptake in plants [6,19]. A previous study of ours showed that the application of different levels of nitrogen fertilizer at different key growth stages mainly affected the metabolism of carbon and nitrogen in late-maturing peach [7]. However, different species of peaches respond differently to the period and level of nitrogen supply.

In the present study, morpho-physiological, transcriptome, and metabolomic aspects of peach fruit at different developmental stages were analyzed, revealing the responses of mid-ripening peach fruit to N availability.

## 2. Materials and Methods

### 2.1. Site Description and Experimental Design

The experiment was conducted between June and July 2019 (summer), at a peach orchard of Institute of Forestry and Pomology (IFP) (116.2° E, 39.97° N), Beijing Academy of Agriculture and Forestry Sciences, Beijing, China. The plant material was peach cultivar “Ruiguang 35” with mid-ripening variety, which was bred by the IFP at 2013 (parents were ‘Fantasy’ × ‘Ruiguang 19′). Trees were planted in April 2007 at a density of 1000 plant/ha (5 m between rows and 2 m between plants, plain). The soil type at the study sites was a calcareous alluvial fluvo-aquic soil with a loamy and silt texture. The physicochemical properties of top 0–20 cm soil were as follows: pH (H_2_O) 7.6, soil organic matter 29.0 g/kg, total N 1.98 g/kg, available phosphorus (P) 11 mg/kg, and available potassium (K) 169 mg/kg.

Nitrogen was applied in the form of urea, which was applied to soil at the fruit pit hardening stage (PH) (5 June 2019) and fruit expansion stage (FE) (1 July 2019), in the form of furrows, with two 20 cm deep trenches dug on both sides of the tree at 2.5 m from the tree, and watered immediately after each application by drip irrigation. At each stage, four N treatments were applied at rates from low to high of 0, 100, 200, and 400 kg N/ha. Treatments were imposed in a random complete block. The orchard climate was a warm temperate semi-humid semi-arid monsoon climate; the average rainfall was 483.9 mm; June, July and August were the rainy months. The detailed fertilization regimes are shown in Table 1.

### 2.2. Plant Sampling and Measurements

After fertilizer application, leaf SPAD values were measured at regular time intervals, at 14 (19 June), 25 (1 July), 31 (7 July), 41 (17 July) and 50 (26 July) days after fertilization at the pit hardening stage, while at 7 (7 July), 10 (17 July) and 19 (26 July) days after fertilization at the fruit expansion stage, ten leaves on each tree were marked and measured using SPAD-502 (Konica Minolta, Tokyo, Japan). After ripening, fruits corresponding to each treatment were picked and weighed, and the yield was estimated. Meanwhile, the samples of fruit pulp were heated at 105 °C for 30 min, dried to a constant weight at 75 °C, and ground into powder. An appropriate amount of the ground plant materials was used to determine total N concentration by a modified Kjeldahl digestion method [20].

The soluble solids content was determined by handheld digital refractometer (PAL-1, Atago, Japan). The skin hardness and peel hardness were measured using a stable micro systems texture analyzer (TA-XT plus, Surrey, UK). Determination of reducing sugars was performed according to the protocol of Gilibowski (2020) [21]. Vitamin C (VC) content was determined with reference to Loong (2021) [22].

### 2.3. Sample Preparation and Metabolite Extraction

The fresh samples were immediately frozen in liquid nitrogen, and the metabolic analysis was performed by Wuhan MetWare Biotechnology Co., Ltd. (Wuhan, China) (www.metware.cn). The freeze-dried sample was crushed using a mixer mill (MM 400, Retsch) with a zirconia bead for 1.5 min at 30 Hz. Around 100 mg of lyophilized powder was extracted with 1.2 mL 70% (*v*/*v*) methanol solution, and vortexed for 30 s every 30 min for 6 times in total. Samples were placed in a refrigerator at 4 °C overnight. Following centrifugation at 12,000 rpm for 10 min, the extracts were filtrated (SCAA-104, 0.22 μm pore size; ANPEL, Shanghai, China, http://www.anpel.com.cn/) before UPLC-MS/MS analysis. The sample extracts were analyzed using an UPLC-ESI-MS/MS system (UPLC, SHIMADZU Nexera X2, www.shimadzu.com.cn/; MS, Applied Biosystems 4500 Q TRAP, www.appliedbiosystems.com.cn/). The analytical conditions were as follows, UPLC, column, Agilent SB-C18 (1.8 µm, 2.1 mm × 100 mm). The mobile phase was consisted of solvent A, pure water with 0.1% formic acid, and solvent B, acetonitrile with 0.1% formic acid. Sample measurements were performed with a gradient program that employed the starting conditions of 95% A, 5% B. Within 9 min, a linear gradient to 5% A, 95% B was programmed, and a composition of 5% A, 95% B was kept for 1 min. Subsequently, a composition of 95% A, 5% B was adjusted within 1.1 min and kept for 2.9 min. The flow velocity was set as 0.35 mL per minute; the column oven was set to 40 °C; the injection volume was 4 μL. The effluent was alternatively connected to an ESI-triple quadrupole-linear ion trap (QTRAP)-MS. LIT and triple quadrupole (QQQ) scans were acquired on a triple quadrupole-linear ion trap mass spectrometer (Q TRAP), an AB4500 Q TRAP UPLC/MS/MS System, equipped with an ESI Turbo Ion-Spray interface, operating in positive and negative ion mode and controlled by Analyst 1.6.3 software (AB Sciex). The ESI source operation parameters were as follows: ion source, turbo spray; source temperature, 550 °C; ion spray voltage (IS), 5500 V (positive ion mode)/−4500 V (negative ion mode); ion source gas I (GSI), gas II (GSII), and curtain gas (CUR) were set at 50, 60, and 25.0 psi, respectively; collision-activated dissociation (CAD) was high. Instrument tuning and mass calibration were performed with 10 and 100 μmol/L polypropylene glycol solutions in QQQ and LIT modes, respectively. QQQ scans were acquired as MRM experiments with collision gas (nitrogen) set to medium. DP and CE for individual MRM transitions were performed with further DP and CE optimization. A specific set of MRM transitions were monitored for each period according to the metabolites eluted within this period. The variable importance in project (VIP) score was obtained by the OPLS-DA model, and metabolites with VIP ≥ 1.0, |Log2 (Fold Change)| ≥ 1 and *p*-value ≤ 0.05 were defined as significantly changed metabolites.

### 2.4. RNA-Seq and Annotation

The total RNA of the peach fruit was isolated using TRIzol reagent (Invitrogen, Carlsbad, CA, USA) for transcriptome analysis, and three technical replicates were performed for each sample. Using 5 μg RNA per sample for library construction, the cDNA libraries were sequenced by Wuhan MetWare Biotechnology Co., Ltd. (www.metware.cn, Wuhan, China) using the Illumina HiSeq platform (Illumina Inc., San Diego, CA, USA). After rapid filtering [23] (version 0.18.0), the clean reads were compared with the apple genome (https://iris.angers.inra.fr/gddh13/index.html) using HISAT2.2.4 and Bowtie2 tools [24,25]. For each transcription region, the values of fragment per kilobase of transcript per million mapped reads (FPKM) were calculated using RESM software. DESeq2 software was used to identify the differentially expressed genes (DEGs) [26] by fold change ≥ 1.5 and divergence probability ≥ 0.8 [27]. Gene Ontology (GO) and the Kyoto Encyclopedia of Genes and Genomes (KEGG) tools were used to analyze the DEGs.

### 2.5. qRT-PCR Analysis

A Revert Aid First Strand cDNA Synthesis Kit (Thermo Scientific, Waltham, MA, USA) was used to reverse transcribe 1 μg RNA with a CFX96 instrument (BioRad, Hercules, CA, USA) and SYBR^®^ Premix Ex Taq™ II (Takara, Dalian, China) to perform quantitative real-time reverse transcriptase PCR (qRT-PCR). The 2^−ΔΔCT^ method was used to compute the relative expression level of each gene. Four biological samples were used in all experiments. The primers used for qRT-PCR are listed in Table S1.

### 2.6. Statistical Analysis

Yield and nitrogen concentration were three replicates and SPAD was four replicates. Fruit quality was determined on the basis of four replicates. Except for FE-N2, which employed five replicates, the metabolites of the other treatments were determined on the basis of six replicates. The transcriptome employed three replicates. The statistical analysis of the data from the control and different plant treatments was performed on the basis of analysis of variance (one-way ANOVA) using SPSS 20.0 software. A probability value of *p* < 0.05 was considered to denote a statistically significant difference.

## 3. Results

### 3.1. Effect of Nitrogen Supply on Yield Attributes and Chlorophyll Contents in Peach Leaves at Different Growth Stages

**Figure 1:**
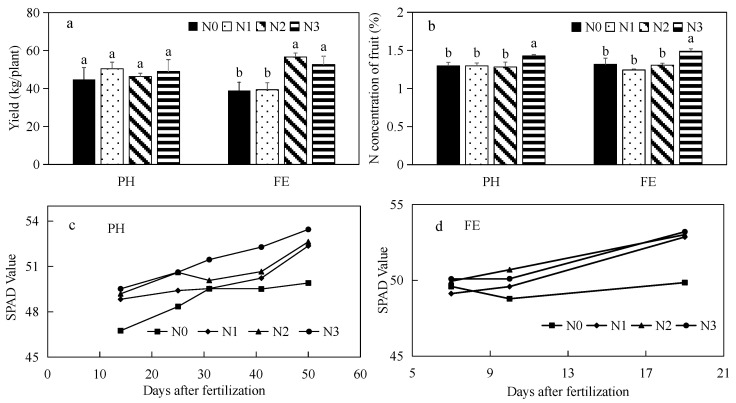
Different effects of nitrogen fertilization on plant growth for application at different growth stages. (**a**) Yield; (**b**) nitrogen (N) concentration of fruit; (**c**,**d**) SPAD value of leaf at different growth stages and for different nitrogen supply levels. PH represents the application of nitrogen fertilizer during the fruit pit hardening stage; FE represents the application of nitrogen fertilizer during the fruit expanding stage. N0, 0 kg N/ha; N1, 100 kg N/ha; N2, 200 kg N/ha; and N4, 400 kg N/ha. Different letters behind the values in the same column indicate significant difference between the treatments at the same growth stage.

Different levels of N fertilizer at the critical growth stage of fruit ripening had different effects on fruit yield. Nitrogen supply at the fruit pit hardening stage (PH) had limited impact on the final yield (Figure 1a). In contrast, N application at the N2 and N3 levels at the fruit expansion stage (FE) yielded 39.60% more on average than that of N1 and N0 treatments. No significant differences in yield were detected between N2 and N3 treatments (Figure 1a). Application of 400 kg N/ha (N3) at both pit hardening and fruit expansion stages resulted in 13.16–19.84% higher N concentrations in fruits at maturity when compared to other N treatments (Figure 1b).

Leaf greenness is estimated by the relative chlorophyll content. Regarding N application at the pit hardening stage, leaf SPAD readings of the N0 plants were always lower than the other three treatments (except on 7 July), and the SPAD values of N3 plants remained the highest and increased with development (Figure 1c). In contrast, there were no significant differences in SPAD readings between N treatments on 7 July. By 17 July, the leaf SPAD values of N2 plants were the highest, and the N0 treatment was the lowest. On 26 July, the SPAD values of the other three treatments were significantly higher than the N0 treatment (Figure 1d).

### 3.2. Effect of Nitrogen Supply on Fruit Quality at Different Growth Stages

Nitrogen application at different stages of fruit development significantly affected the fruit quality (Table 2). Compared to the N0 treatment, N1 application at FE markedly increased soluble solid content of fruit by 9.80%, while no significant differences were detected between N applications at PH (Table 2). The nitrogen fertilizer had no significant impact on fruit peel hardness, regardless of N application timing.

However, fertilizer application at the pit hardening stage affected the fruit peel hardness. The peel hardness with the N3 treatment increased by 22.46% compared to with the N1 treatment. Nitrogen application of 400 kg/ha (N3) at the pit hardening stage significantly increased the reducing sugar in the fruit by 9.16% relative to the N0. However, N application at the expansion stage had no significant effect on the reducing sugar of the fruit. Nitrogen application at the pit hardening stage had limited effects on the VC content of the fruit. However, excessive application of nitrogen fertilizer (i.e., the N3 treatment) at the fruit expansion stage significantly reduced fruit VC content by 39.05–48.09% relative to the other N treatments (Table 2).

### 3.3. Metabolite Profiles of Fruit in Response to Nitrogen Availability

**Figure 2:**
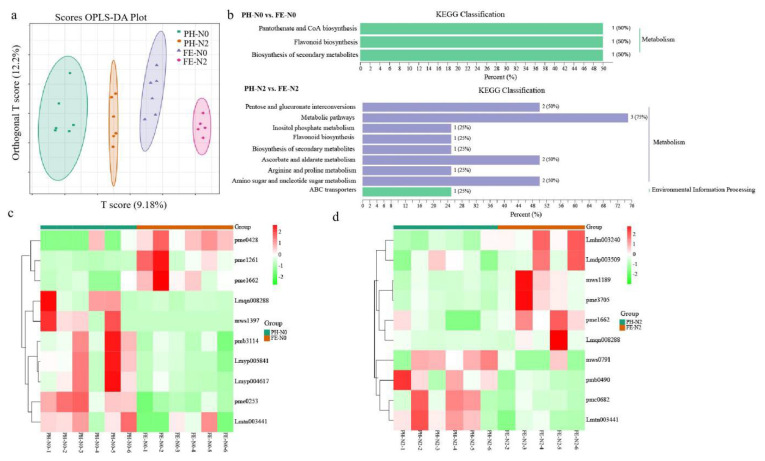
Different effects of nitrogen fertilization on metabolomic for application at different growth stages. (**a**) OPLS-DA; (**b**) KEGG classification; (**c**,**d**) heat map of different metabolites in peach fruit under different growth stages and different nitrogen supply levels. The values are means of six biological replicates (except FE-N2). PH-N0, 0 kg N/ha application at the fruit pit hardening stage; PH-N2, 200 kg N/ha application at the fruit pit hardening stage; FE-N0, 0 kg N/ha application at the fruit expansion stage; FE-N2, 200 kg N/ha application at the fruit expansion stage.

To investigate the changes in fruit metabolites during fruit development stages with different N levels, analyses of differentially accumulated metabolites (DAMs) in PH-N0 vs. FE-N0 and PH-N2 vs. FE-N2 were performed. Orthogonal partial least-squares discriminant analysis (OPLS-DA) showed a 9.18% T score and 12.2% orthogonal T score (Figure 2a). It was observed that all biological replicates were grouped, indicating high reliability of the resulting metabolome data (Figure 2c, top). For each N supply, strong developmental impacts were found in fruit metabolites. The PH-N0 vs. FE-N0 metabolites were mapped to the KEGG database, and most DAMs were associated with metabolism (Figure 2b). The fundamental biological pathways involved in PH-N0 vs. FE-N0 were pantothenate and CoA biosynthesis, flavonoid biosynthesis, and biosynthesis of secondary metabolites. The KEGG classification involved in PH-N2 vs. FE-N2 included metabolism and environment information processing. Significantly enriched metabolites were identified in pentose and glucuronate interconversions, metabolic pathway, inositol phosphate metabolism, flavonoid biosynthesis, secondary metabolites biosynthesis, ascorbate and aldarate metabolism, arginine and proline metabolism, amino sugar and nucleotide sugar metabolism, and ABC transporters (Figure 2b).

Using VIP ≥ 1.0 and |Log2 (Fold Change)| ≥ 1 as a threshold for significant differences, there were 10 differentially expressed substances in PH-N0 vs. FE-N0 in fruit, and these were divided into five categories: flavonoids, phenolic acids, amino acids and derivatives, lipids, and saccharides and alcohols (Table 3). Compared to PH-N0, 5,4′-dihydroxy-7-methoxyflavanone in fruit increased 3.7 times in FE-N0, while chrysin-7-O-glucoside, pinocembrin-7-O-glucoside, epicatechin gallate, and epicatechin–epiafzelechin decreased by 2.3, 2.9, 773.3, and 2.1 times, respectively. One phenolic acid in the fruit increased and the other one decreased in PH-N2 vs. FE-N2. 3,4,5-trimethoxycinnamic acid increased 3.84 times more than PH-N0 in the FE-N0, while 2-acetyl-3-hydroxyphenyl-1-O-glucoside decreased by 2.09 times. N-acetyl-L-leucine (the amino acid and derivatives) and 3-hydroxyoctadecanoic acid of lipids significantly decreased by 2.05 and 2.78 times, respectively. In addition, D-panthenol of saccharides and alcohols significantly increased 5.31-fold. In PH-N2 vs. FE-N2, there were six types of metabolites in fruits, including flavonoids, phenolic acids, alkaloids, organic acids, lipids, saccharides, and alcohols (Table 4). Compared to PH-N2, two of flavonoids in FE-N2 were upregulated, and one metabolite was reduced. In the significantly increased flavonoids metabolites, 5,4′-Dihydroxy-7-methoxyflavanone and Quercetin-3-O-xyloside reached more than two times the significant difference level. The poncirin content was downregulated 3-fold in PH-N2 vs. FE-N2. Similar to PH-N0 vs. FE-N0, the 2-Acetyl-3-hydroxyphenyl-1-O-glucoside content was significantly reduced in PH-N2 vs. FE-N2. The 4-aminoindole and p-coumaroylputrescine contents decreased 2.98- and 2.3-fold, respectively. Among the upregulated substances, D-galacturonic acid, 3-hydroxyoctadecanoic acid, and D-glucuronic acid have increased by 2.70, 2.13, and 2.63 times, respectively.

### 3.4. Impact of Nitrogen on Differentially Expressed Genes in Fruit

**Figure 3:**
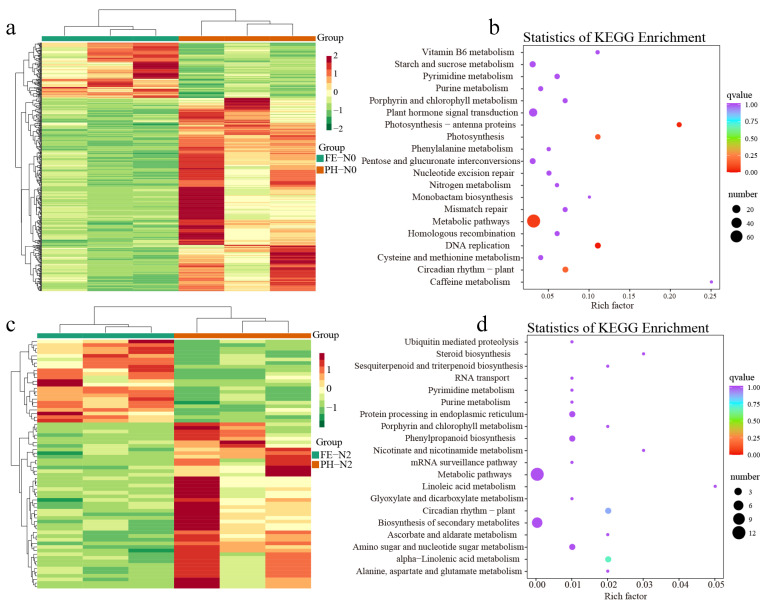
Different effects of nitrogen fertilization on the transcriptome for application at different growth stages. (**a**,**c**) Cluster heat map; (**b**,**d**) statistics of KEGG enrichment of different genes in peach fruit at different growth stages and under different nitrogen supply levels. PH-N0, 0 kg N/ha application at the fruit pit hardening stage; PH-N2, 200 kg N/ha application at the fruit pit hardening stage; FE-N0, 0 kg N/ha application at the fruit expansion stage; FE-N2, 200 kg N/ha application at the fruit expansion stage.

To compare the gene expression levels at the pit hardening and expansion stages under the same N application levels, the profiles of fruit transcriptomes were analyzed by RNA-Seq. According to the DESeq2 analysis |log2 (Fold Change)| ≥ 1 and false discovery rate (FDR) < 0.05, 441 transcripts were differentially expressed in PH-N0 vs. FE-N0, including 98 genes upregulated and 343 downregulated (Table S2). This was in agreement with the fact that transcription changes more at the PH stage than at the FE stage in fruit (Figure 3a,c). GO enrichment analysis indicated that the greatest proportion of DEGs was enriched in biological process in fruits of different development stages. The enrichment analyses of the top 20 pathways showed that most identified DEGs involved in the metabolic pathways, N metabolism, and biosynthesis of secondary metabolites at PH-N0 vs. FE-N0 in peach fruit (Figure 3b). GO annotation of the DEGs found 23 upregulated and 46 downregulated genes in PH-N2 vs. FE-N2 (Table S3). These differential genes were annotated to the top 20 KEGG pathways, including biological process, cellular component, and molecular function (Figure 3d).

**Figure 4:**
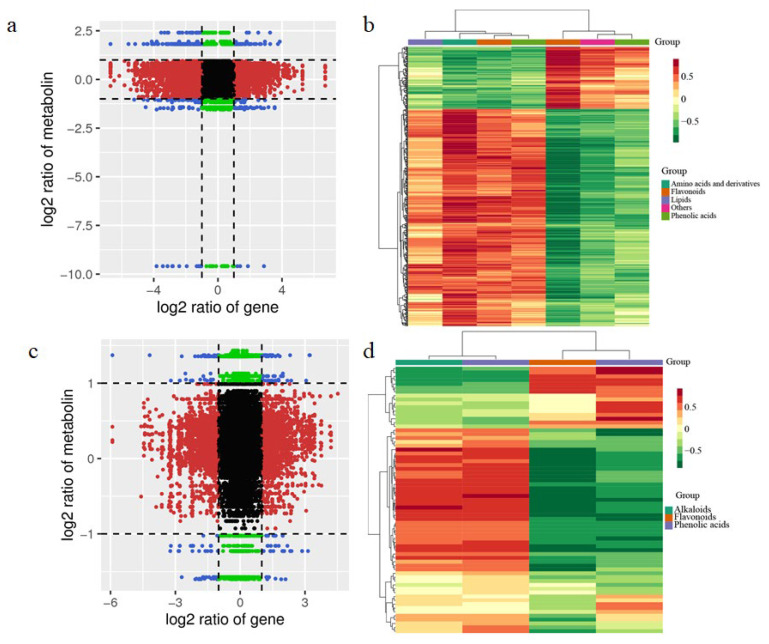
The conjoint analysis of transcriptome and metabolome analyzed the effects of nitrogen application at different growth stages. (**a**,**c**) Associations of transcriptomic and metabolomic variation quadrant diagrams in PH-N0 vs. FE-N0 and PH-N2 vs. FE-N2; the differential thresholds are indicated as black dotted lines. Outside the threshold lines, there are instead differences in gene/metabolites, and within the threshold lines are indicated the unchanged gene/metabolites. Each point shows a gene/metabolite. Black dots, green dots, red dots, and blue dots indicate unchanged genes/metabolites, differentially accumulated metabolites with unchanged genes, differentially expressed genes with unchanged metabolites, and both differentially expressed genes and differentially accumulated metabolites, respectively. (**b**,**d**) Heat maps of the correlation coefficient clusters (>0.8) (for interpretation of the colors in this figure, the reader is referred to the web version of this article).

Figure 4a,c shows the Pearson’s correlation coefficients for the nine quadrants. The differential expression patterns of gene and metabolite were consistent in the third and seventh quadrants. Genes were positively related to the regulation of metabolites. DEGs and DAMs with Pearson’s correlation coefficients (PCCs) higher than 0.8 were further selected and are represented as a heat map (Figure 4b,d).

### 3.5. Profiles of DEGs and DAMs in Flavonoid Biosynthetic Pathways in Developing Fruits

**Figure 5:**
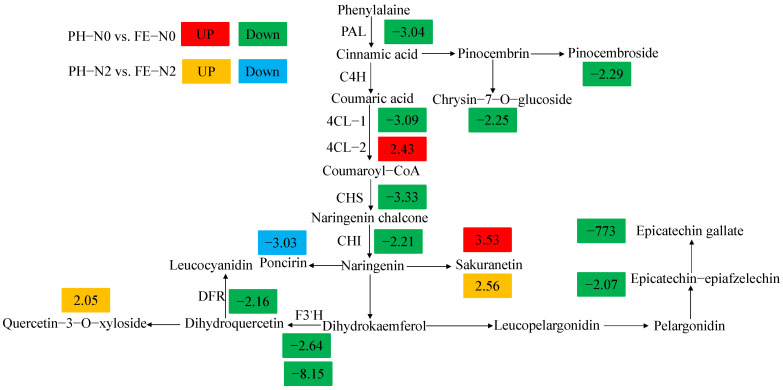
Differentially expressed genes and differentially accumulated metabolites associated with flavonoid metabolism pathways. PAL, phenylalaninammo nialyase; 4CL, 4-Coumarate,coenzyme A ligase; CHS, chalcone synthase; CHI, chalcone isomerase; F3′H, flavonoid 3′-hydroxylase; DFR, dihydroflavonol 4-reductase. The numbers in the red box represent multiples of upregulation of genes and metabolites, and the green box represented multiples of downregulation of genes and metabolites at PH-N0 vs. FE-N0. The numbers in the orange box represent multiples of upregulation of genes and metabolites, and the blue box represents multiples of downregulation of genes and metabolites at PH-N2 vs. FE-N2. PH-N0, 0 kg N/ha application at the fruit pit hardening stage; PH-N2, 200 kg N/ha application at the fruit pit hardening stage; FE-N0, 0 kg N/ha application at the fruit expansion stage; FE-N2, 200 kg N/ha application at the fruit expansion stage.

The expression of six structural genes of the flavonoid biosynthesis pathway (PAL, 4CL, CHS, CHI, F3′H, and DFR) plays a critical role in anthocyanin biosynthesis. Seven differentially expressed genes at PH-N0 vs. FE-N0. PpPAL (*Prupe.2G211800*) expression was 3 times lower in the hard pit stage than in the fruit expanding stage under N0 treatment (Figure 5). Similarly, the expression of CHI (*Prupe.2G263900*), and DFR (*Prupe.4G200500*) were significantly downregulated by 2.21 and 2.16 times, respectively. Two 4CL genes were singled out at PH-N0 vs. FE-N0; *Prupe.1G232500* was downregulated 3.09-fold, while *Prupe.5G154000* was upregulated 2.43-fold. The *F3′H Prupe.5G077600* and *Prupe.1G580300* showed an upregulation trend of 2.64- and 8.15-fold at PH-N0 vs. FE-N0. Among the different metabolites at PH-N0 vs. FE-N0, Epicatechin gallate was significantly downregulated (Figure 5).

### 3.6. Validation of DEGs by qRT-PCR

To validate the RNA-seq results, we used qRT-PCR for expression analysis of ten peach genes. The RT-qPCR analysis results were not significantly different from the RNA-Seq data, and similar trends were found in the up- and downregulated genes (Figure S1). These results confirmed the reliability of the RNA-Seq results and reflected.

## 4. Discussion

### 4.1. Effects of Application of Nitrogen Fertilizer at Different Growth Stages on Yield and Fruit Quality of Peach

Nitrogen deficiency significantly affects plant growth and physiological development, causing yield losses and low quality [6]. Nitrogen stress often results in a decrease in plant biomass, leaf nitrogen content, chlorophyll content, and photosynthetic rate of different varieties of olives [28]; similar results have previously also been found in apple [6], rice [9], wheat [29], and soybean [1]. In addition to the dose, the application times and management adopted in the culture can also affect the availability of the applied N [30,31]. There were significant differences in the sensitivity of plants to N fertilizer at different developmental stages. Both N application rates and timing significantly affected the nutritional growth of mango tree, e.g., the number of branches, branch length, fruit count, stem biomass [12]. Low N significantly increased lycopene content in tomato at different developmental stages [32]. For Rosaceae fruit trees, many changes in physiological and molecular processes occur during fruit development. Different timings and methods of N application modify N dynamics in apple and could have differential effects on fruit color, fruit set, and yield [33,34]. It has been shown that the application of an appropriate amount of N fertilizer at different times could improve soluble solids and fruit skin color of peach fruit [35].

In the current study, different N levels at the fruit expansion stage significantly affected yield, but N application was not associated with the yield at the PH stage (Figure 1a). During the hardening period, fruit growth rate is relatively slow, and fruit N demands are met through the remobilization of stored reserves and new acquisitions [36]. Therefore, this period is not sensitive to the amount of nitrogen applied and does not affect the yield (Figure 1a). During the fruit expansion period, the fruit requires a large amount of N to maintain rapid growth, and is sensitive to N availability. The present study results demonstrated that N application at different fertility stages affected peach yield and fruit quality, including soluble solids, skin hardness, reducing sugar, and VC (Table 2). However, N application at this stage (FE) did not affect the fruits’ pulp firmness, which was in agreement with the results previously found in temperate fruit tree species [35]. Regardless of the N application levels, the saccharides and alcohols in fruits at the expansion stage were significantly increased compared to the pit hardening stage, indicating that the expansion stage is the key stage of fertilization and could effectively improve fruit quality.

### 4.2. Effects of Application of Nitrogen Fertilizer at Different Growth Stages on Transcriptome and Metabolome of Peach

Higher plants can synthesize many compounds that differ in terms of chemical structure and biological activity. Fruits contain many metabolites responsible for their organoleptic properties, nutritional value, and pharmaceutical activities [13]. Peach represents a particular model fruit in terms of physiology, anatomy, and metabolism, and is different from other model fruits, such as tomato, strawberry, or grape. 

The composition and richness of amino acids are the key indexes of nutritional quality, and are also important determinants of flavor [37]. The current study identified 79 amino acids in fruit at PH-N0 vs. FE-N0 (Table S4). One amino acid, N-Acetyl-L-leucine, was significantly decreased at PH-N0 vs. FE-N0 (Table 3). These results suggest that differences in amino acid composition and abundance are crucial for changes in flavor matter.

Alkaloids are a kind of basic organic compound containing N. Alkaloids, homologous metabolites of terpenoids, are compounds unique to *Zanthoxylum* fruits, and have high economic value in the food and pharmaceutical industries [38,39,40]. Meanwhile, some alkaloids have antibacterial properties. The accumulation of p-Coumaroylputrescine in the rosette leaves of *A. thaliana* suggests their involvement in the plant’s defense responses [41]. Recently, the accumulation of p-coumaroylputrescine was demonstrated in rice cells treated with a strong fungal elicitor [42]. Because of the robustness of this response, and the simple detection of p-coumaroylputrescine by LC-MS/MS, this metabolite can be used as a reliable marker to monitor rice defense responses [43,44,45]. When N supply was adequate, e.g., N2, application at the core hardening stage eventually increased p-coumaroylputrescine content in the fruit, indicating improved fruit defense responses.

The accumulation of organic acids improves plants’ ability to adapt to hostile environments [1]. Organic acids can play a role in maintaining the intracellular ion balance to adapt to alkaline stress [46]. The fruit’s flavor components are generally related to the contents of sugar, organic acids, and phenols [47]. Both D-galacturonic acid and D-glucoronic acid are related to pectin formation, which is one of the main components of the plant cell wall and is an important part of the growing cell plate during cell division, and are significantly upregulated in PH-N2 vs. FE-N2 treatment. This may be due to the rapid cell enlargement during fruit enlargement and the accumulation of pectin precursors in fruit.

Flavonoids exist widely in the plant community, and these are closely related to the formation of flower color, UV protection, resistance to pathogens, and plant growth regulation [48]. In a previous study, the effects of N on the flavonoid pathway were extensively examined [6]. Meanwhile, the flavonoid synthesis pathway was highly responsive to fruit development, which has been proven in other plants as well, like Chinese jujube [49], apricot [50], and banana [51]. In the present study, five flavonoids (chrysin-7-O-glucoside, pinocembrin-7-O-glucoside, epicatechin gallate, epicatechin-epiafzelechin, poncirin) were decreased in the expansion stage (Figure 5). In the synthetic pathway of flavonoids, the relevant synthetic genes were reduced after fertilization, regardless of N availability (Figure 5). The present study results indicate that the fruits are susceptible to disease during the expansion period, since flavonoids are positively related to disease resistance of fruits. For the present peach species, fertilization during fruit expansion increased the sakuranetin content in the fruits regardless of N supply levels. Studies have shown that sakuranetin is related to plant photosynthesis [52], indicating that peach fruit grows rapidly during fruit expansion, requiring more photosynthates.

## 5. Conclusions

In summary, the effects of nitrogen application at different fruit development stages on peach fruit physiology, transcriptome, and metabolites were analyzed. Limited N availability influenced plant growth and physiological characteristics. Different growth stages also influenced DEGs and DAMs in peach. Peach responded to the different environments through regulating flavonoid pathways. These results provide a broader and better understanding of the metabolic processes underlying the N availability and its developmental impacts on mid-ripening peach fruit.

## Figures and Tables

**Table 1:** The specific fertilization scheme.

Treatment Name	N Supply (kg N/ha)	Urea (g)	KH_2_PO_4_ (g)	KCl (g)
N0	0	0	77	124
N1	100	267	77	124
N2	200	534	77	124
N3	400	1070	77	124

**Table 2:** Quality index of fruit under different growth stages and different nitrogen supply levels.

Quality Index	N Application at the Fruit Pit Hardening Stage (PH)				N Application at the Fruit Expansion Stage (FE)			
	N0	N1	N2	N3	N0	N1	N2	N3
Soluble solid (%)	15.51 ± 0.27 a	15.20 ± 0.76 a	15.16 ± 0.29 a	15.62 ± 0.10 a	13.99 ± 0.26 b	15.36 ± 0.52 a	14.25 ± 0.22 ab	14.07 ± 0.54 ab
Skin hardness (kg/cm^2^)	4.25 ± 0.33 ab	3.67 ± 0.32 b	4.44 ± 0.34 ab	5.34 ± 0.47 a	4.22 ± 0.34 a	4.66 ± 0.32 a	4.96 ± 0.32 a	4.20 ± 0.46 a
Peel hardness (kg/cm^2^)	2.71 ± 0.13 ab	2.36 ± 0.16 b	2.67 ± 0.16 ab	2.89 ± 0.13 a	2.70 ± 0.15 a	2.81 ± 0.18 a	2.75 ± 0.12 a	2.68 ± 0.20 a
Reducing sugar (%)	2.73 ± 0.11 b	2.83 ± 0.05 ab	2.79 ± 0.04 ab	2.98 ± 0.13 a	3.24 ± 0.22 a	3.12 ± 0.18 a	3.28 ± 0.07 a	3.03 ± 0.16 a
VC (mg/100 g)	8.00 ± 0.18 a	7.47 ± 0.83 a	7.77 ± 0.24 a	8.13 ± 0.65 a	9.44 ± 0.46 a	9.09 ± 0.25 a	8.04 ± 0.69 a	4.90 ± 0.62 b

Different letters following the values in the same row indicate significant differences between the N treatments at each growth stage.

**Table 3:** Differentially accumulated compounds with VIP (variable importance in projection) ≥ 1, and |Log2 (Fold Change)| ≥ 1 as for upregulation/downregulation in ‘RG35′ PH-N0 vs. PE-N0. PH-N0, 0 kg N/ha application at the fruit pit hardening stage; FE-N0, 0 kg N/ha application at the fruit expansion stage.

Component Name	Metabolite Name	Treatment		VIP	Log2FC	Type
		PH-N0	FE-N0			
Flavonoids	5,4′-Dihydroxy-7-methoxyflavanone (Sakuranetin)	1.26 × 10^3^	4.71 × 10^3^	13.9	18.2	up
	Chrysin-7-O-glucoside	4.03 × 10^5^	1.79 × 10^5^	17.1	11.7	down
	Pinocembrin-7-O-glucoside (Pinocembroside)	1.25 × 10^5^	4.27 × 10^4^	16.8	15.5	down
	Epicatechin gallate	6.96 × 10^3^	9.00 × 10	17.1	95.9	down
	Epicatechin-epiafzelechin	8.66 × 10^4^	4.19 × 10^4^	16.0	10.5	down
Phenolic acids	3,4,5-Trimethoxycinnamic acid	1.81 × 10^3^	6.96 × 10^3^	17.7	19.5	up
	2-Acetyl-3-hydroxyphenyl-1-O-glucoside	1.74 × 10^4^	8.31 × 10^3^	14.7	10.7	down
Amino acids and derivatives	N-Acetyl-L-leucine	3.28 × 10^4^	1.60 × 10^4^	21.1	10.4	down
Lipids	3-Hydroxyoctadecanoic Acid	4.28 × 10^5^	1.54 × 10^5^	13.9	14.8	down
Saccharides and Alcohols	D-Panthenol	6.67 × 10^3^	3.54 × 10^4^	18.8	24.1	up

**Table 4:** Differentially accumulated compounds with VIP (variable importance in projection) ≥ 1, and |Log2 (Fold Change)| ≥ 1 as for upregulation/downregulation in ‘RG35′ PH-N2 vs. FE-N2. PH-N2, 200 kg N/ha application at the fruit pit hardening stage; FE-N2, 200 kg N/ha application at the fruit expansion stage.

Component Name	Metabolite Name	Treatment		VIP	Log2FC	Type
		PH-N2	FE-N2			
Flavonoids	5,4′-Dihydroxy-7-methoxyflavanone (Sakuranetin)	1.38 × 10^3^	3.53 × 10^3^	13.8	13.6	up
	Quercetin-3-O-xyloside (Reynoutrin)	8.66 × 10^3^	1.77 × 10^4^	11.7	10.3	up
	Poncirin (Isosakuranetin-7-O-neohesperidoside)	4.00 × 10^3^	1.32 × 10^3^	12.6	16.0	down
Phenolic acids	Benzoylmalic acid	2.51 × 10^5^	6.49 × 10^5^	19.7	13.7	up
	2-Acetyl-3-hydroxyphenyl-1-O-glucoside	1.99 × 10^4^	6.61 × 10^3^	13.4	15.9	down
Alkaloids	4-Aminoindole	2.28 × 10^5^	7.65 × 10^4^	14.3	15.7	down
	p-Coumaroylputrescine	6.02 × 10^4^	2.58 × 10^4^	16.1	12.3	down
Organic acids	D-Galacturonic acid	5.56 × 10^5^	1.50 × 10^6^	18.9	14.3	up
Lipids	3-Hydroxyoctadecanoic acid	1.78 × 10^5^	3.79 × 10^5^	11.8	10.9	up
Saccharides and Alcohols	D-Glucoronic acid	6.23 × 10^5^	1.64 × 10^6^	19.1	14.0	up

## Data Availability

Date related to this article can be found, in the online version, at doi: 10.6084/m9.figshare.20486589.

## Supplementary Materials

The following supporting information can be downloaded at: https://www.mdpi.com/article/10.3390/metabo12100905/s1, Figure S1: The relative expression of ten peach genes; Table S1: Sequences of primers used in this study, Table S2: Transcriptomic results at PH-N0 vs. FE-N0, Table S3: Transcriptomic results at PH-N2 vs. FE-N2, Table S4: 79 amino acid results at PH-N0 vs. FE-N0.
