# Supplementary material for: Transcriptome and Metabolome Studies on Pre-Harvest Nitrogen Impact on Fruit Yield and Quality of Peach (Prunus persica L.)
Source: Metabolites. 2022 Sep 26;12(10):905. doi: 10.3390/metabo12100905 (PMC9608177; doi:10.3390/metabo12100905)
Supplement: Supplementary file 1 [file metabolites-12-00905-s001.zip › metabolites-1921835-supplementary.pdf]

SUPPORTING INFORMATION

Table S1. Sequences of primers used in this study.

Gene Name	Forward Primer	Reverse Prime
Prupe.8G14300	CGGGCTAATGTCGGTCCAGAAAC	ACGAGCAGCCAACAGTGTAACAAG
Prupe.6G141100	GATCAGTGCAGCAATGGAGGGTTAG	TCGCCACAGTCAACAAAGTCGTAG
Prupe.1G503100	TGCTGTGCTACAACTGTCTTCATCC	GTGCTTCACTCCCTCAACCTTTCC
Prupe.6G287600	ACACTGGAGACAACACCATCATTGC	CGTGAAGAATGACCACAACAGCAAC
Prupe.4G165800	CACCACAACACAAACAAACGCCTAG	CAAAGCAAACCAGCAGCAGCAG
Prupe.6G110800	CTACCCACACATTGCCTCACACAG	TCTGATGCCTGAGTAGCCTTGGAG
Prupe.2G211800	CTACCCACACATTGCCTCACACAG	TCTGATGCCTGAGTAGCCTTGGAG
Prupr.3G032200	CCACCTCCTACACCTACTCCTTCTC	CAAGCCTGCCGTTGATCCTCTC
Prupe.3G032200	ACCGTCCTCCACACTTACTCTACTG	GCGGACTTGCTCGGGGATAATG
Prupe.2G246800	GGCACCTCCTTCTGGAACTTCTTC	AGCACGGCAGGCGATATGATAAC
ACTIN	TGAAGGAGAGGGAAGGTGAAAG	GGTGTGACGATGAAGAGTGATG

Table S2. Transcriptomic results at PH-N0 vs. FE-N0.

ID	PH-N0-1	PH-N0-2	PH-N0-3	FE-N0-1	FE-N0-2	FE-N0-3	
Prupe.1G013900	42.37	14	18.31	9.23	8.41	13	up
Prupe.1G019400	6.22	8.85	5.82	29.02	24.46	14.07	down
Prupe.1G021400	1812.16	1097.02	1529.46	374.78	227.15	702.16	up
Prupe.1G021800	488.05	99.57	80.99	30.26	16.51	44.44	up
Prupe.1G034800	4.43	3.19	6.73	2.09	1.75	2.16	up
Prupe.1G042300	5.74	3.35	5.14	2.38	1.81	2.62	up
Prupe.1G042500	6.16	24.44	15.03	40.47	34.9	34.61	down
Prupe.1G045800	11.77	8.08	10.94	3.6	2.2	6.64	up
Prupe.1G052400	3	7.12	3	1.49	2.39	1.48	up
Prupe.1G052600	4.44	1.56	2.34	0.52	0.48	1.13	up
Prupe.1G057500	30.81	16.81	23.66	8.96	7.72	17.09	up
Prupe.1G076800	2.86	7.01	3.12	9.69	8.42	8.13	down
Prupe.1G103000	7.91	13.1	8.79	24.59	22.15	17.26	down
Prupe.1G104900	5.45	2.59	3.47	0.67	0.49	1.03	up
Prupe.1G105800	439.68	971.48	527.72	1462.07	1611.01	1170.44	down
Prupe.1G137000	92.72	67.88	67.65	29.1	30.62	49.55	up
Prupe.1G137200	192.37	153.22	153.55	70.08	73.75	61.65	up
Prupe.1G137300	4.43	7.59	4.32	12.22	10.19	12.14	down
Prupe.1G141000	390.04	593.96	609.54	1183.47	1739.17	1086.18	down
Prupe.1G143100	1.77	0.87	1.44	0.64	0.45	0.75	up
Prupe.1G146400	8.57	33.33	17.83	77.9	44.32	41.82	down
Prupe.1G162200	0.92	1.01	0.53	1.74	1.63	2.7	down
Prupe.1G178400	1.76	0.74	2.08	0.45	0.14	0.68	up
Prupe.1G191100	7.72	11.99	8.36	21.78	27.79	13.4	down
Prupe.1G201700	8.15	5.17	7.4	3.21	2.96	2.73	up
Prupe.1G204700	0.12	0.9	0.33	1.31	2.56	1.7	down
Prupe.1G210900	4.16	4.47	2.07	0.45	0.76	1.56	up
Prupe.1G217200	1.44	2.48	4.06	0.68	1.15	1.13	up
Prupe.1G218500	26.33	9.75	8.71	2.96	2.97	7.95	up
Prupe.1G222000	12.53	7.13	8.84	3.22	5.29	4.85	up
Prupe.1G224500	156.67	151.58	151.13	56.95	77.78	71.75	up
Prupe.1G230500	4.73	2.48	5.43	0.23	0.3	1.39	up
Prupe.1G232500	31.77	14.8	22.02	7.15	6.88	8.13	up
Prupe.1G233000	92.08	223.77	115.59	352.01	331.85	194.46	down
Prupe.1G233700	3.96	1.9	6.11	1.15	0.87	2.07	up
Prupe.1G239900	4.73	3.77	4.63	0.43	0.43	0.58	up
Prupe.1G243500	46.39	43.33	56.42	121.46	99.67	74.86	down
Prupe.1G273100	10.91	4.29	14.71	3.59	2.47	5.5	up
Prupe.1G276700	547.29	221.22	972.9	18	6.82	73.21	up
Prupe.1G297500	6.48	5.07	5.34	1.93	2.84	3.22	up
Prupe.1G301900	0.51	1.89	0.5	2.86	3.74	3.03	down
Prupe.1G305500	1.79	0.87	0.79	0.36	0.29	0.25	up

Prupe.1G309900	18.78	4.77	10.67	1.65	1.36	4.11	up
Prupe.1G311800	77.74	64.67	74.38	35.1	27.79	34.82	up
Prupe.1G312200	1.34	1.2	1.63	3.03	3.47	2.37	down
Prupe.1G320600	4.67	2.22	6.95	1.11	0.45	1.92	up
Prupe.1G326400	42.72	56.07	45.31	21.89	13.96	27.44	up
Prupe.1G336400	2.08	0.91	1.14	0.38	0.2	0.44	up
Prupe.1G337500	4.24	9.31	7.3	17.92	15.05	12.84	down
Prupe.1G339700	6.03	2.53	3.5	0.2	0.45	0.12	up
Prupe.1G341500	4.19	4.66	1.73	0.68	0.34	0.93	up
Prupe.1G341800	12.45	9.43	11.4	4.72	7.56	3.81	up
Prupe.1G343500	20.74	11.85	22.31	6.49	6.62	12.96	up
Prupe.1G347200	3.78	1.74	2.75	1.4	1.18	1.26	up
Prupe.1G348000	2.44	0.71	0.82	0.29	0.34	0.44	up
Prupe.1G350700	23.91	11.27	30.23	2.29	1.65	10	up
Prupe.1G371600	127.82	64.6	64.47	27.77	32.93	41.9	up
Prupe.1G372800	55.36	25.24	46.14	9.46	7.93	20.25	up
Prupe.1G375700	20.24	22.05	10.5	6.72	3.97	7.02	up
Prupe.1G377700	1.4	1.05	0.98	0.19	0.54	0.4	up
Prupe.1G379800	9.35	4.5	8.79	2	1.85	3.43	up
Prupe.1G380600	10.58	5.59	23.35	1.67	1.72	6.32	up
Prupe.1G381200	2.65	0.72	4.66	0.39	0.2	0.93	up
Prupe.1G390800	54.49	34.92	16.8	5.27	6.67	10.8	up
Prupe.1G413500	24.14	23.33	26.54	10.52	9.67	10.13	up
Prupe.1G418100	14.59	5.79	6.22	3.24	2.96	3.9	up
Prupe.1G420700	8.89	4.02	3.69	1	1.02	1.4	up
Prupe.1G425100	22.98	13.85	12.07	5.89	3.42	4.92	up
Prupe.1G430300	31.08	6.07	7.83	1.01	1.76	2.03	up
Prupe.1G434100	0.3	1.71	0.16	0.13	0.13	0.04	up
Prupe.1G445000	2.41	1.39	2.47	0.52	0.88	1.12	up
Prupe.1G447400	4.17	1.59	2	0.63	1	1.05	up
Prupe.1G451700	69.51	102.32	103.4	201.04	208.96	135.48	down
Prupe.1G461400	35.86	20.25	41.22	8.6	7.65	20.51	up
Prupe.1G462200	2.2	2.89	3.05	0.63	0.72	1.02	up
Prupe.1G462400	13.72	9.36	11.89	2.35	2.74	3.74	up
Prupe.1G478400	20.94	30.75	26.23	10.69	8.11	9.95	up
Prupe.1G490600	60.75	46.52	47.3	21.7	24.02	21.71	up
Prupe.1G493300	5.51	3.44	5.19	1.69	2.06	3.01	up
Prupe.1G494200	10.4	2.92	3.4	1.26	0.47	1.81	up
Prupe.1G494800	22.72	17.21	20.37	10.63	9.54	9.03	up
Prupe.1G498000	10.01	4.26	8.06	2.62	2.75	3.33	up
Prupe.1G503100	0.47	0.62	0.35	1.12	4.63	1.17	down
Prupe.1G503400	2.25	3.81	4.25	9.38	6.12	7.46	down
Prupe.1G505400	39.2	24.65	18.9	8.86	7.78	10.67	up
Prupe.1G528600	88.6	56.28	66.19	32.05	26.22	40.72	up

Prupe.1G557800	49.75	16.95	19.24	4.3	6.39	9.64	up
Prupe.1G561600	51.14	70.88	56.14	103.96	139.89	125.66	down
Prupe.1G573600	126.6	26.37	28.18	9.2	12.11	13.72	up
Prupe.1G577800	438.29	324.33	449.09	98.96	115.89	197.89	up
Prupe.1G579300	3	3.16	3.49	1.89	1.53	1.21	up
Prupe.1G580300	2.21	0.87	0.59	0.21	0.05	0.19	up
Prupe.2G000500	1.86	2.73	1.7	3.89	6.73	3.35	down
Prupe.2G017800	480.7	224.93	267.04	55.89	69.91	130.57	up
Prupe.2G025100	17.49	23.22	31.72	8.43	5.36	12.11	up
Prupe.2G039300	862.46	821.85	612.87	359.87	359.09	234.63	up
Prupe.2G065900	30.72	17.69	22.29	10.25	13.28	7.68	up
Prupe.2G071800	37.89	20.26	26.67	6.93	9.18	9.08	up
Prupe.2G072000	129.97	165.59	96.34	349.05	360.41	156.09	down
Prupe.2G079900	2.54	2.22	2.15	6.13	5.57	2.88	down
Prupe.2G098700	7.99	18.74	13.82	32.35	22.19	29.23	down
Prupe.2G107600	11.12	9.73	11.22	4.14	3.48	4.85	up
Prupe.2G111900	11.18	10	16.06	4.14	3.15	5.87	up
Prupe.2G124300	39.44	31.78	25.71	10.37	15.55	16.62	up
Prupe.2G138600	9.8	6.25	11.08	3.83	2.82	5.89	up
Prupe.2G154500	13.76	10.43	10.07	5.48	6.9	3.34	up
Prupe.2G164400	342.18	169.59	185.76	85.3	122.13	92.13	up
Prupe.2G168300	4.84	2.41	4.44	0.62	1.9	1.47	up
Prupe.2G178100	177.56	83.72	88.04	54.11	42.05	68.22	up
Prupe.2G180700	9.76	4.97	4.75	2.05	2.69	3.34	up
Prupe.2G195300	9.54	1.84	2.27	0.39	0.58	1.04	up
Prupe.2G198500	5.23	7.8	7.21	2.62	2.3	2.31	up
Prupe.2G200400	5.32	9.95	10.16	4.97	2.38	3.71	up
Prupe.2G202500	14.14	13.84	10.85	4.04	3.03	6.3	up
Prupe.2G202600	7.65	11.99	4.5	0.94	0.38	2.92	up
Prupe.2G207400	0.65	4.55	1.96	11.99	17.1	4.3	down
Prupe.2G207500	0.22	1.72	1.19	8.92	10.83	1.73	down
Prupe.2G211800	1.59	5.27	6.93	1.4	1.01	2.13	up
Prupe.2G212200	23.82	19.43	27.47	10.65	10.4	11.19	up
Prupe.2G217400	1.35	2.49	1.45	5.19	5.04	2.89	down
Prupe.2G224700	7.41	3.73	3.92	1.09	1.37	1	up
Prupe.2G230000	23.91	19.46	37.7	76.38	88.7	53.01	down
Prupe.2G232000	17.78	8.64	21.98	1.18	1.8	4.36	up
Prupe.2G232200	5.07	2.93	6.34	0.23	0	0.42	up
Prupe.2G239400	2.2	1.11	3.06	0.67	0.77	0.63	up
Prupe.2G243800	153.14	190.34	177.31	295.69	462.96	268.48	down
Prupe.2G246800	3.36	22.34	0.31	0.12	0.71	0.23	up
Prupe.2G252500	9.89	4.76	5.87	2.4	2.19	3.46	up
Prupe.2G262400	296.86	280.92	220.96	658.65	787.8	270.36	down
Prupe.2G263300	24.69	6.79	7.38	1.99	1.74	2.47	up

Prupe.2G263600	1541.94	1200.97	2138.41	608.67	509.16	763.23	up
Prupe.2G263900	16.79	14.14	9.73	6.79	5.64	6	up
Prupe.2G266100	1.89	4.12	3	9.8	11.48	7.84	down
Prupe.2G275500	0.87	1.43	0.99	0.4	0.48	0.47	up
Prupe.2G279700	5.59	2.04	1.01	0.04	0.04	0.46	up
Prupe.2G280800	149.18	149.93	138.75	59.68	43.86	83.25	up
Prupe.2G283900	57.81	26.46	32.53	13.05	15.22	17.1	up
Prupe.2G295300	66.51	33.62	21.22	13.58	10.33	21.04	up
Prupe.2G311900	70.33	47.2	76.73	29.84	27.76	32.58	up
Prupe.2G315600	12.23	8.05	9.99	3.72	4.05	4.84	up
Prupe.2G318800	25.35	30.02	19.76	49.47	69.01	44	down
Prupe.2G319600	3.8	4.48	5.74	1.6	1.26	2.2	up
Prupe.2G321300	16.48	8.39	15.24	6.06	4.84	7.52	up
Prupe.2G322300	23.81	33.15	54.95	13.77	19.5	13.5	up
Prupe.2G323500	3.96	0.55	0.92	0.07	0.07	0.34	up
Prupe.3G012400	150.02	117.81	113.96	179.39	460.04	258.83	down
Prupe.3G013700	0.4	1.25	0.54	2.61	3.04	2.03	down
Prupe.3G025900	2.72	7.89	1.07	0.95	1.48	0.75	up
Prupe.3G031700	6.35	1.21	3.1	0.21	0.39	0.88	up
Prupe.3G032200	93.18	52.18	50.75	3.82	5.82	11.96	up
Prupe.3G038500	34.3	17.66	29.21	9.03	10.15	13.31	up
Prupe.3G038900	0.54	1.12	1.29	2.32	3.06	2.97	down
Prupe.3G039000	38	27.81	39.74	12.94	10.57	26.21	up
Prupe.3G051100	4.61	4.82	6.81	9.4	14.84	7.85	down
Prupe.3G055000	3.03	0.56	3.95	0.45	0.48	0.62	up
Prupe.3G074800	923.25	598.32	815.22	220.6	220.84	475.79	up
Prupe.3G077200	90.54	45.73	68.15	33.76	26.75	36.72	up
Prupe.3G113500	33.91	25.3	36.03	10.72	9.56	19.45	up
Prupe.3G114200	55.35	21.77	24.43	6.31	9.76	9.65	up
Prupe.3G117500	0.64	0.59	0.73	1.77	2.33	1.14	down
Prupe.3G132500	11.26	3.74	16.54	1.47	1.3	4.02	up
Prupe.3G149600	290.37	248.16	169.93	107.35	110.03	38.62	up
Prupe.3G149700	246.71	252.58	254.87	125.18	107.91	76.11	up
Prupe.3G167800	0.39	0.49	0.57	1.05	0.91	1.03	down
Prupe.3G172600	54.56	35.7	44.57	16.27	17.86	20.59	up
Prupe.3G174700	6.23	1.68	2.28	0.13	0.13	0.32	up
Prupe.3G179600	10.36	7.62	11.31	2.83	4.15	4.78	up
Prupe.3G180400	11.93	9.16	14.61	5.21	3.29	4.87	up
Prupe.3G187300	184	254.16	211.26	513.08	569.77	382.7	down
Prupe.3G189400	3.17	2.64	2.1	1.14	0.94	1.7	up
Prupe.3G202500	54.58	33.42	42.47	12.16	10.48	14.73	up
Prupe.3G210700	9.17	3.4	4.07	0.92	1.18	1.7	up
Prupe.3G219100	0	0.32	0.21	2.63	16.58	0.84	down
Prupe.3G230200	7.83	3.69	4.16	1.35	1.56	1.88	up

Prupe.3G232400	286.56	211.24	243.85	111.67	101.88	83.89	up
Prupe.3G232700	1.5	3.37	2.35	4.93	5.05	4.69	down
Prupe.3G239900	23.62	20.22	14.65	7.77	6.07	13.22	up
Prupe.3G241700	0.68	3.53	1.37	0.44	0.43	0.4	up
Prupe.3G242700	6.94	3.53	4.76	1.93	1.71	3.08	up
Prupe.3G249200	15.87	21.98	28.32	4.52	3.77	10.84	up
Prupe.3G253800	13.82	8.6	9.06	3.6	2.61	5.9	up
Prupe.3G261300	16.17	2.68	5.94	1.61	1.87	3	up
Prupe.3G261600	26.73	15.17	16.62	8.66	8.69	8.11	up
Prupe.3G266900	28.34	17.47	18.92	5.53	5.93	9.76	up
Prupe.3G269300	2.37	1.69	1.62	0.1	0.81	0.63	up
Prupe.3G269400	3.79	3.76	3.44	1.17	0.69	2.62	up
Prupe.3G278300	12	22.17	13.73	28.76	47.33	25.18	down
Prupe.3G283600	12.33	4.57	13.15	2.96	2.51	6.17	up
Prupe.3G283800	17.95	8.08	11.79	4.94	4.16	5.03	up
Prupe.3G285300	192.14	149.7	102.71	71.62	50.65	70.94	up
Prupe.3G285400	55.46	37.91	39.81	18.38	15.81	23.97	up
Prupe.3G291000	2.19	0.52	3.08	0.27	0.16	0.68	up
Prupe.3G300900	8.45	14.66	5.9	2.89	2.5	5.77	up
Prupe.3G301600	2.42	1.49	5.81	0.76	0.86	1.46	up
Prupe.3G302600	0.24	0.4	0.68	1.29	0.88	1.29	down
Prupe.3G306600	30.49	12.84	30.4	1.64	2.03	6.79	up
Prupe.3G312900	2.3	4.49	1.5	0.84	1.23	0.74	up
Prupe.4G012500	10.19	10.7	6.11	3.34	5.95	3.18	up
Prupe.4G024500	13.8	4.21	1.75	0.9	0.71	1.84	up
Prupe.4G034500	2.14	3.85	4.01	6.07	9.02	4.95	down
Prupe.4G039000	1.02	0.78	1.01	0.37	0.23	0.31	up
Prupe.4G039900	13.58	21.89	20.95	39.18	59.31	26.33	down
Prupe.4G040900	23.81	12.74	19.6	5.75	7.47	7.86	up
Prupe.4G051200	12.33	6.35	8.2	3.69	4.19	4.75	up
Prupe.4G054300	59.84	27.76	72.51	6.67	7.04	17.02	up
Prupe.4G059600	0.75	1.76	1.38	4.28	5.81	2.59	down
Prupe.4G061900	19.63	35.11	22.25	66.08	69.68	49.51	down
Prupe.4G063700	3.46	4.82	4.33	1.87	1.68	2.41	up
Prupe.4G069400	2.07	4.42	3.3	11.33	13.52	4.86	down
Prupe.4G069600	4.98	5.41	4.74	12.29	13.08	9.51	down
Prupe.4G076300	2.8	3.92	1.08	0.46	1	0.56	up
Prupe.4G077900	8.25	6.97	7.88	2.89	2.83	4.57	up
Prupe.4G086300	15.05	14.91	12.35	33.26	33.16	25.62	down
Prupe.4G096300	1.45	0.84	1.57	0.06	0.18	0.47	up
Prupe.4G098300	2.98	1	0.7	0.12	0.11	0.25	up
Prupe.4G120400	7.64	0.82	7.66	0.24	0.05	0.91	up
Prupe.4G150500	43.99	18.52	26.36	8.95	8.83	15.25	up
Prupe.4G151600	112.89	54.1	113.52	30.43	25.08	44.22	up

Prupe.4G155300	1.35	0.75	2.33	0.17	0.03	0.42	up
Prupe.4G155900	5.52	3.59	6.07	2.01	1.45	3.8	up
Prupe.4G158000	2.79	5.22	1.94	6.64	10.62	4.78	down
Prupe.4G159700	2.89	23.6	8.24	1.41	3.16	2.92	up
Prupe.4G165800	0.88	1.07	0.44	3.25	4.34	0.94	down
Prupe.4G169000	1.95	1.6	1.18	4.9	8.36	2.47	down
Prupe.4G171300	0.96	1.13	0.96	0.51	0.42	0.39	up
Prupe.4G191200	3.87	0.25	0.5	0.02	0.06	0.05	up
Prupe.4G200500	57.2	35.61	53.67	22.2	18.7	26.85	up
Prupe.4G201900	16.27	22.84	16.26	45.48	37.93	31.01	down
Prupe.4G212300	2.28	1.75	2.67	0.77	0.74	1.09	up
Prupe.4G214800	12.07	7.11	9.12	3.41	3.85	4.93	up
Prupe.4G220700	1.8	1.38	1.16	2.97	2.37	3.17	down
Prupe.4G220800	26.31	21.38	54.83	9.79	11.34	15.28	up
Prupe.4G222100	1.35	4.33	1.13	0.48	0.43	0.48	up
Prupe.4G224700	77.91	62	70.35	29.49	30.96	36.29	up
Prupe.4G225300	1.67	5.33	0.64	0.3	0.55	0.37	up
Prupe.4G244100	27.09	20.09	16.42	3.26	3.07	5.31	up
Prupe.4G248000	45.15	20.46	30.72	14.54	12.27	15.44	up
Prupe.4G249500	57.69	55.64	72.38	17.13	12.61	31.12	up
Prupe.4G255600	1.21	0.96	0.28	2.13	5.66	1.36	down
Prupe.4G276500	5.61	3.98	3.47	0.78	0.6	1.78	up
Prupe.5G014600	1.76	3.19	2.89	4.85	6.03	4.76	down
Prupe.5G015000	23.67	17.57	32.04	9.3	9.44	16.85	up
Prupe.5G015500	318.97	306.13	237.14	119.07	105.29	192.34	up
Prupe.5G018300	8.91	7.47	5.12	19.38	13.93	12.83	down
Prupe.5G020000	1.06	2.71	2.22	6.18	5.8	3.15	down
Prupe.5G042300	10.4	7.35	16.77	4.16	3.26	7.3	up
Prupe.5G061800	5.89	6.39	1.96	0.48	2.41	0.38	up
Prupe.5G062300	9.88	7.34	5.35	2.32	4.03	4.48	up
Prupe.5G064800	2.73	7.46	6.85	23.67	42.87	13.38	down
Prupe.5G072000	18.31	3.71	5.2	2.13	1.09	1.19	up
Prupe.5G072700	38.23	21.06	19.22	6.77	3.74	10.95	up
Prupe.5G072800	7660.02	6170.98	6147.52	3059.09	2456.9	2850.43	up
Prupe.5G073900	7.91	5.37	5.55	0.89	1.39	2.07	up
Prupe.5G076800	20.98	16.55	14.37	4.63	7.54	9.01	up
Prupe.5G076900	3.81	3.39	1.48	0.39	1.61	0.4	up
Prupe.5G077000	8.68	6.25	4.96	14.81	16.98	7.8	down
Prupe.5G077600	3.17	2.28	1.79	0.86	0.46	1.42	up
Prupe.5G080300	13.33	13.34	11.49	4.72	4.87	5.28	up
Prupe.5G101300	92.3	58.74	76	33.08	25.81	37.14	up
Prupe.5G101400	21.72	6.17	5.76	2.86	2.98	3.04	up
Prupe.5G104300	29.87	11.07	11.32	2.98	4.29	3.38	up
Prupe.5G109300	1.14	2	0.96	0.25	0.37	0.37	up

Prupe.5G117200	7.08	4.47	8.69	2.23	2.87	3.57	up
Prupe.5G117700	11.6	9.75	17.28	5.15	4.45	8.32	up
Prupe.5G120500	4.91	1.39	2.27	0.45	0.64	1.13	up
Prupe.5G126200	152.71	169.45	162.17	55.54	54.22	43.04	up
Prupe.5G134000	14.61	4.76	8.8	2.69	2.04	4.26	up
Prupe.5G134800	38.49	39.03	34.75	18.91	16.07	17.61	up
Prupe.5G140900	1.83	1.19	1.97	0.16	0.22	0.49	up
Prupe.5G148500	4.33	8.57	7.33	1.49	4.23	1.94	up
Prupe.5G149500	130.47	109.88	114.3	36.69	47.97	47.98	up
Prupe.5G154000	6.67	8.25	4.93	15.23	22.35	10.75	down
Prupe.5G156300	4.83	3.04	5.65	2.31	2.05	2	up
Prupe.5G159900	6.87	9.33	7.61	3	3.67	2.7	up
Prupe.5G161600	15.16	6.04	13.67	3.54	1.75	3.71	up
Prupe.5G163800	3.75	4.6	2.15	0.24	1.88	0.69	up
Prupe.5G169500	7.39	3.62	3.5	1.56	1.77	2.04	up
Prupe.5G178100	9.7	3.79	7.57	0.69	0.55	2.14	up
Prupe.5G194300	13.02	5.53	4.98	1.64	2.02	2.18	up
Prupe.5G195200	0.66	4.49	0.66	0.11	0.22	0.03	up
Prupe.5G205700	15.55	5.69	20.95	1.53	1.23	4.27	up
Prupe.5G206100	18.56	6.23	6.47	0.47	1.85	1.24	up
Prupe.5G211000	3.09	1.07	4.68	0.67	0.52	1.62	up
Prupe.5G216500	39.8	9.46	23.72	4.53	8.98	6.05	up
Prupe.5G219700	45.95	31.32	32.69	17.23	12.13	22.87	up
Prupe.5G231000	15.3	4.84	8.24	2.98	4.45	3.44	up
Prupe.5G239000	8.19	6.24	1.7	1.24	2.38	1.35	up
Prupe.5G241000	0.28	0.31	0.35	0.89	1.05	0.62	down
Prupe.5G243500	8.44	9.84	6.29	19.19	24.07	10.67	down
Prupe.6G002700	6.1	3.51	4.21	1.42	1.97	2.43	up
Prupe.6G012100	55.79	21.37	52.75	11.89	12.56	21.51	up
Prupe.6G016200	27.49	14.46	37.42	3.21	3.39	10.95	up
Prupe.6G021100	32.56	15.33	35.94	2.77	2.17	10.8	up
Prupe.6G021700	9.12	4.46	14.63	0.16	0.79	2.04	up
Prupe.6G026200	5.62	5.19	3.98	1.83	1.51	3.17	up
Prupe.6G028000	0.83	1.48	1.84	3	4.75	2.54	down
Prupe.6G032400	175.58	171.05	138.14	64.06	45.1	116.78	up
Prupe.6G041500	0.2	1.11	0	3.53	4.03	2.36	down
Prupe.6G042000	6.05	2.36	7.89	0.71	0.56	2.68	up
Prupe.6G043300	11.19	20.91	7.8	46.17	46.07	25.72	down
Prupe.6G052800	4.09	4.27	4.22	2.02	1.43	1.77	up
Prupe.6G053900	18.3	15.38	15.09	3.33	3.76	3.73	up
Prupe.6G054800	185.75	182.28	214.24	71.82	84.39	109.66	up
Prupe.6G055600	52.72	28.42	46.46	14.44	13.46	23.71	up
Prupe.6G059600	2.9	1.91	1.9	0.69	0.68	1.26	up
Prupe.6G060100	3.61	1.04	2.96	0.35	0.32	0.85	up

Prupe.6G060300	18.51	2.2	4.84	0.83	1.37	1.71	up
Prupe.6G077200	7.75	8.96	11.95	22.5	21.84	14.91	down
Prupe.6G081300	29.49	9.92	35	2.8	5.89	3.8	up
Prupe.6G085300	2.91	4.53	5.87	10.22	7.44	15.62	down
Prupe.6G089500	12.54	8.45	10.36	5.15	3.43	5.8	up
Prupe.6G097700	38.3	22.59	31.03	7.52	4.58	13.17	up
Prupe.6G102300	7.47	6.18	7.85	2.66	2.92	3.67	up
Prupe.6G108800	119.89	84.54	99.06	34.38	35.51	54.84	up
Prupe.6G110800	10.65	17.85	20.16	55.61	49.02	50.25	down
Prupe.6G119100	7.13	9.88	7.01	31.55	18.52	21.17	down
Prupe.6G141100	90.09	322.6	280.83	1292.37	1930.13	901.58	down
Prupe.6G149100	0.7	1.11	0.7	1.96	2.43	1.76	down
Prupe.6G157800	4.54	3.65	5.07	11.91	11.93	8.25	down
Prupe.6G159900	61.6	35.52	60.37	18.19	13.81	31.53	up
Prupe.6G161600	1161.11	919.12	1273.84	388.96	309.03	446.84	up
Prupe.6G173300	8.92	7.45	9.34	1.6	0.71	1.36	up
Prupe.6G175300	15.88	10.22	11.96	3.65	3.06	5.78	up
Prupe.6G196000	1.71	1.25	1.3	0.21	0.8	0.24	up
Prupe.6G213800	2.76	8.02	5.56	12.46	16.22	11.74	down
Prupe.6G219000	29.6	16.52	15.64	7.78	5.96	10.79	up
Prupe.6G223000	0.65	0.68	1.77	0.29	0.3	0.41	up
Prupe.6G225300	40.59	29.37	13.93	7.14	9.5	10.49	up
Prupe.6G229300	13.34	7.25	10.52	3.8	3.28	6.54	up
Prupe.6G236500	0.5	1.12	0.75	3.3	3.4	1.42	down
Prupe.6G237000	1.95	0.84	1.72	0.36	0.36	0.38	up
Prupe.6G237300	2.46	2.05	1.47	0.57	0.79	0.95	up
Prupe.6G241500	0.88	0.44	0.95	0.07	0.07	0.32	up
Prupe.6G251000	1.1	4.86	2.16	7.38	12.91	5.01	down
Prupe.6G251900	27.93	24.45	33.76	10.93	15.12	13.55	up
Prupe.6G252500	484.32	448.95	610.05	225.96	165.86	343.48	up
Prupe.6G255100	219.4	468.8	310.93	405.35	1077.36	647.21	down
Prupe.6G261300	26.31	21.63	24.04	4.05	3.97	7.25	up
Prupe.6G264500	160.84	75.18	99.53	34.24	26.12	52.25	up
Prupe.6G264800	5.59	1.74	2.7	0.66	1.16	1.49	up
Prupe.6G265300	2.14	2.63	2.64	0.84	1.22	0.99	up
Prupe.6G267400	3.26	2.3	4.43	1.41	0.96	1.86	up
Prupe.6G268400	85.06	46.87	37.54	12.97	9.15	23.13	up
Prupe.6G270300	3.52	1.83	2.24	0.68	0.67	1.14	up
Prupe.6G274500	21.85	8.82	14.74	6.56	4.94	5.94	up
Prupe.6G277500	153.16	196.7	80.7	61.86	69.31	67.8	up
Prupe.6G278100	21.67	14.57	11.4	2.15	6.98	6.63	up
Prupe.6G287600	6.89	5.72	5.54	16.13	29.97	22.36	down
Prupe.6G288400	9.31	9.71	15.44	5.21	4.33	5.4	up
Prupe.6G290100	0.91	3.03	0.8	6.69	5.19	4.75	down

Prupe.6G296500	13.05	15.08	17.58	35.71	36.46	21.29	down
Prupe.6G296800	1.98	3.79	3.02	7.54	8.69	3.68	down
Prupe.6G301600	2.8	0.86	1.26	0.52	0.37	0.84	up
Prupe.6G304600	146.06	154.06	213.4	54.92	59.3	128.85	up
Prupe.6G305700	1.34	2.46	1.47	0.66	0.79	0.79	up
Prupe.6G307600	44.44	46.56	25.18	84.86	92.52	51.76	down
Prupe.6G312100	0.32	0.96	1.9	4.24	5.52	3.03	down
Prupe.6G312400	3.96	1.02	2.62	0.55	0.24	0.94	up
Prupe.6G322000	24.49	9.01	25.16	1.55	1	4.01	up
Prupe.6G323700	73.87	45.78	82.79	26.11	27.45	32.48	up
Prupe.6G340600	1.85	0.96	1.65	0.52	0.44	0.73	up
Prupe.6G360500	32.12	16.18	30.43	10.67	12.16	14.34	up
Prupe.6G364900	8.81	7.45	4.28	0.52	1.88	2.18	up
Prupe.7G015100	3.68	6.87	2.15	0.69	0.7	0.99	up
Prupe.7G022500	14.08	5.75	8.08	3.65	5.16	4.16	up
Prupe.7G040900	3.97	2.79	3.26	0.86	0.6	0.24	up
Prupe.7G041600	11.74	1.33	5.98	0.88	0.56	2.01	up
Prupe.7G049600	1.92	0.85	2.42	0.61	0.41	0.75	up
Prupe.7G061600	17.66	15.89	25.27	7.61	5.63	14.22	up
Prupe.7G063100	7.72	2.65	6.06	2.57	1.74	3.18	up
Prupe.7G068000	7.73	4.78	6.08	2.51	2.36	2.78	up
Prupe.7G085300	3.75	9.3	7.65	3.72	3.29	1.79	up
Prupe.7G086800	130.27	123.27	119.64	54.66	64.5	45.9	up
Prupe.7G087200	91.42	62.32	66.55	24.69	26.63	42.22	up
Prupe.7G087400	7.46	1.07	3.17	0	0.11	0.25	up
Prupe.7G102100	21.34	14.58	32.35	7.79	10.73	10	up
Prupe.7G107600	0.74	1.15	0.95	1.46	3.36	1.99	down
Prupe.7G108800	11.04	4.78	12.39	2.56	2.87	4.73	up
Prupe.7G120500	0.54	1.17	0.52	0.13	0.19	0.11	up
Prupe.7G122500	1.53	3.23	1.24	6.06	6.35	3.68	down
Prupe.7G133700	34.7	12.38	16.25	10.87	8.32	10.67	up
Prupe.7G136300	3.6	3.96	3.69	6.43	12.94	12.25	down
Prupe.7G149100	39.97	12.65	14.6	5.56	7.34	9.89	up
Prupe.7G150000	0.66	0.71	0.66	1.34	1.87	1.41	down
Prupe.7G162200	3.8	2.94	3.92	1.44	1.55	1.73	up
Prupe.7G169500	1.8	1.71	1.49	0.39	0.41	1.04	up
Prupe.7G170100	33.93	36.57	23.58	75.23	109.69	35.04	down
Prupe.7G183100	0.56	0.51	0.96	0.27	0.27	0.33	up
Prupe.7G187600	114.25	92.94	98.75	46.45	40.3	49.95	up
Prupe.7G190300	114.55	145.16	106.82	54.03	28.82	90.79	up
Prupe.7G192600	95	82.36	109.06	34.23	38.72	60.47	up
Prupe.7G197100	3.62	4.39	3.8	1.02	1.46	1.28	up
Prupe.7G223400	0.52	1.22	1.63	3.84	2.54	2.15	down
Prupe.7G250800	39.97	87.26	81.5	142.62	228.81	125.87	down

Prupe.7G253800	54.34	76.1	58.39	21.77	22.93	36.16	up
Prupe.7G259600	857.55	629.56	743.79	267.22	244.24	441.97	up
Prupe.7G264000	4.84	1.26	1.47	0.08	0.42	0.46	up
Prupe.8G023900	0.56	1.57	1.12	3.77	5.06	2.68	down
Prupe.8G039100	99.93	123.2	155.77	38.67	38.58	80.03	up
Prupe.8G042000	17.5	7.65	14.39	4.08	3.33	6.68	up
Prupe.8G066200	15.86	13.57	8.8	1.74	1.18	5.3	up
Prupe.8G081300	12.77	0.47	1.46	0.05	0.16	0.34	up
Prupe.8G083600	5.16	2.83	3.49	1.12	1.17	2	up
Prupe.8G083700	51.08	42.6	53.79	22.73	22.63	19.1	up
Prupe.8G085100	5.34	3.08	3.86	1.76	2.19	1.61	up
Prupe.8G093900	25.28	13.08	19.18	7.98	4.6	12.04	up
Prupe.8G101500	10.83	16.63	21.8	4.2	4.21	5.55	up
Prupe.8G125500	98.53	116.94	89.25	200.12	260.95	206.88	down
Prupe.8G134400	14.25	10.1	9.75	3.03	3.39	2.85	up
Prupe.8G141900	305.78	265.93	266.18	132.82	137.73	134.79	up
Prupe.8G142700	3.63	2.05	1.48	0.56	1.02	0.3	up
Prupe.8G143000	0.13	0.79	0.48	4.15	3.79	1.41	down
Prupe.8G148400	10.65	11.75	8.96	23.21	34.85	18.5	down
Prupe.8G151000	14.23	10.86	14.75	5.78	4.27	6.98	up
Prupe.8G157800	10.02	5.99	7.93	1.96	1.53	3.31	up
Prupe.8G163200	7.69	5.52	9.28	2.65	2.76	3.35	up
Prupe.8G164000	96.21	45.74	84.04	11.14	10.11	25.19	up
Prupe.8G164100	27.66	22.11	25.27	10.83	7.8	15.27	up
Prupe.8G165600	417.14	272.97	584.69	114.92	138.75	200.88	up
Prupe.8G168200	63.64	52.59	60.61	25.76	24.73	28.61	up
Prupe.8G179300	76.05	29.07	37.95	10.99	7.89	27.85	up
Prupe.8G181100	2.61	1.68	1.8	0.58	0.5	0.63	up
Prupe.8G185800	0.19	0.37	0.31	0.74	0.67	0.66	down
Prupe.8G188200	19.2	16.78	22.86	10.18	9.66	8.01	up
Prupe.8G191900	55.59	24.84	19.22	7.7	16.78	13.15	up
Prupe.8G196900	13.99	4.11	14.46	2.43	2.13	5.88	up
Prupe.8G198600	150.25	122.52	131.86	64.7	68.07	63.78	up
Prupe.8G209100	0.39	1.35	0.92	3.97	2.72	2.32	down
Prupe.8G210800	12.36	7.4	11.65	4.13	4.58	4.28	up
Prupe.8G215000	4.24	1.66	2.57	1.08	1.5	1.42	up
Prupe.8G215700	100.97	30.31	65.49	9.95	7.01	24.61	up
Prupe.8G216600	3.63	2.96	3.51	0.93	1.14	1.01	up
Prupe.8G224500	17.39	13.37	6.38	2.43	1.67	7.12	up
Prupe.8G233400	1.07	0.23	1.09	0.05	0.05	0.25	up
Prupe.8G235600	12.38	1	2.36	0.3	0.33	1.11	up
Prupe.8G238300	91.82	229.15	157.3	390.13	426.56	198.46	down
Prupe.8G250000	16.96	9.25	27.33	1.2	2.3	4.62	up
Prupe.8G252500	4.91	1.95	2.29	1.02	1.55	1.26	up

Prupe.8G253200	52.32	43.01	54.92	21.12	29.73	20.58	up
Prupe.8G261400	33.39	17.1	23.68	5.11	7.39	7.95	up
Prupe.8G272000	14.45	8.09	22.13	3.94	2.75	8.98	up

Table S3. Transcriptomic results at PH-N2 vs. FE-N2.

ID	PH-N2-1	PH-N2-2	PH-N2-3	FE-N2-1	FE-N2-2	FE-N2-3	
Prupe.1G021800	43.83	96.16	58.55	29.66	19.19	39.79	up
Prupe.1G103600	22.18	11.85	8.95	48.25	25.49	38.32	down
Prupe.1G137000	69.46	128.61	112.44	62.99	45.71	43.02	up
Prupe.1G165700	9.18	8.48	9.94	5.6	3.49	4.71	up
Prupe.1G189800	16.75	8.01	15.58	34.72	17.96	34.52	down
Prupe.1G210900	3.48	5.78	3.78	1.49	1.05	2.09	up
Prupe.1G322500	33.37	51.25	43.02	25.81	15.04	23.08	up
Prupe.1G341800	6.63	5.17	6.36	11.47	10.64	15.05	down
Prupe.1G418100	8.46	14.85	10.72	2.86	5.02	4.02	up
Prupe.1G472800	2.19	1.39	0.59	0.14	0.15	0.39	up
Prupe.1G486600	3.17	5.69	5.58	2.59	2.4	2.12	up
Prupe.1G494200	3.75	4.68	3.48	1.2	2.04	1.15	up
Prupe.1G498600	9.16	14.66	29.44	39.75	62.57	58.54	down
Prupe.2G017800	239.65	291.14	529.22	129.97	204.25	150.24	up
Prupe.2G039300	397.46	397.66	387	904.52	967.31	735.24	down
Prupe.2G124300	19.05	13.47	12.4	37.12	32.8	31.96	down
Prupe.2G192600	3.26	1.88	3.42	4.87	6.19	6.44	down
Prupe.2G200400	6.3	15.5	10.22	2.62	5.91	4.3	up
Prupe.2G202500	11.71	28.87	9.9	7.03	3.84	5.89	up
Prupe.2G243800	157.02	136.78	215.44	318.34	453.75	258.66	down
Prupe.2G305200	216.84	305.07	83.19	78.09	80.17	62.22	up
Prupe.2G320300	5.44	13.85	17.76	4.47	5.07	5.6	up
Prupe.3G039200	2.68	6.14	5.31	1.53	2.36	1.99	up
Prupe.3G057600	0.79	0.68	1.13	2.56	2.02	2.78	down
Prupe.3G107200	23.9	38.12	19.25	16.91	12.51	10.84	up
Prupe.3G114200	11	13.25	14.6	31.36	43.14	40.63	down
Prupe.3G219100	0.15	0.07	0.31	0.34	3.63	1.66	down
Prupe.3G222000	1.6	5.53	1.83	0.64	0.64	0.68	up
Prupe.3G224900	0.62	0.55	1.18	1.89	1.7	1.64	down
Prupe.3G239900	25.99	30.59	30.27	14.11	12.63	14.79	up
Prupe.3G266900	10.97	27.66	26.43	11.22	11.26	7.93	up
Prupe.3G278900	29.84	75.95	53.61	20.12	23.97	23.93	up
Prupe.3G285300	142.08	202.3	131.1	69.53	91.25	66.86	up
Prupe.3G285400	47.7	72.63	40.65	25.96	25.12	21.96	up
Prupe.4G042500	3.65	3.19	4.64	2.27	1.53	1.04	up
Prupe.4G049600	0.2	0.31	1.32	3.13	6.62	7.3	down
Prupe.4G074400	3.8	5.49	4.4	1.87	2.66	2.11	up

Prupe.4G098300	0.56	1.45	1.34	0.29	0.23	0.28	up
Prupe.4G276500	4.96	10.78	5.65	4.13	1.91	1.79	up
Prupe.5G015500	233.51	414.68	386.49	127	218.03	157.26	up
Prupe.5G020000	1.49	1.18	1.21	3.16	2.88	4.01	down
Prupe.5G052300	5.72	16.68	8.22	4.25	5.13	2.76	up
Prupe.5G123200	48.36	141.99	67.23	41.29	36.24	32.09	up
Prupe.5G202800	0.43	3.5	1.23	0.36	0.32	0.38	up
Prupe.5G211200	104.59	184.91	110.96	58.76	75.96	62.53	up
Prupe.5G232300	9.49	29.56	36.92	9.14	5.94	8.01	up
Prupe.6G053900	8.8	6.61	6.66	17.09	14.62	16.74	down
Prupe.6G063700	0.73	0.88	1.57	0.24	0.24	0.49	up
Prupe.6G103900	13.69	8.96	7.5	30.67	19.35	30.83	down
Prupe.6G194100	1.69	6.05	4.05	1.06	0.7	0.43	up
Prupe.6G194200	2.51	2.98	0.78	0.37	0.59	0.28	up
Prupe.6G210200	109.54	152.61	141.56	51.71	92.63	55.29	up
Prupe.6G255100	175.23	209.73	353.59	338.85	580.72	601.37	down
Prupe.6G268400	54.54	66.29	29.21	20.53	12.27	21	up
Prupe.6G364900	8.79	6.61	4.25	3.8	1.46	2.59	up
Prupe.7G015100	5.42	4.35	6.92	2.34	1.41	2.25	up
Prupe.7G016700	3.3	5.75	9.84	1.85	2.51	3.07	up
Prupe.7G139200	0.28	0.35	0.3	1.93	1.05	2.13	down
Prupe.8G030800	3.39	2.05	1.9	5.52	6.35	7.55	down
Prupe.8G066500	1.53	2.16	0.15	0.24	0.12	0.06	up
Prupe.8G109000	1.96	1.15	1.19	3.16	2.99	3.33	down
Prupe.8G127600	9.05	5.13	8.7	14.43	11.71	20.48	down
Prupe.8G178000	2.1	8.29	8.01	0.78	1.71	1.05	up
Prupe.8G201100	19.43	11.31	17.77	38.97	45.04	32.85	down
Prupe.8G224500	16.06	28.48	13.98	10.15	5.97	5.86	up
Prupe.8G235600	2.5	3.22	2.58	1.04	0.98	0.27	up
Prupe.8G243100	0.38	0.46	0.37	1.16	3.16	1.15	down
Prupe.8G263800	4.81	10.9	4.86	2.87	2.39	3.54	up
Prupe.I000900	2.39	3.22	1.56	0.75	0.8	0.8	up

Table S4 79 amino acid results at PH-N0 vs. FE-N0

Index	Compounds	PH-N0	FE-N0	Type
pme0253	N-Acetyl-L-leucine	3.28×10^4	1.60×10^4	down
pme1419	L-Methionine methyl ester	2.61×10^4	3.64×10^4	insig
mws0282	L-Tryptophan	1.47×10^5	2.46×10^5	insig
mws1375	Nicotianamine	2.35×10^4	3.68×10^4	insig
pme1210	L-Methionine	9.65×10^4	1.35×10^5	insig
mws0230	L-Threonine	3.61×10^5	5.45×10^5	insig
mws5037	L-Alanyl-L-leucine	2.09×10^4	3.25×10^4	insig
mws0250	L-Tyrosine	3.65×10^6	5.46×10^6	insig

mws0736	N-Glycyl-L-leucine	3.41×10^4	4.55×10^4	insig
Lmbn000121	3-Cyano-L-alanine	2.11×10^4	2.91×10^4	insig
mws0258	L-Isoleucine	1.21×10^7	1.92×10^7	insig
pme0014	L-Glutamic acid	8.82×10^6	6.99×10^6	insig
mws0227	L-Leucine	1.23×10^7	1.89×10^7	insig
mws5041	L-Glycyl-L-isoleucine	3.27×10^4	4.37×10^4	insig
Zmjp000182	N-Monomethyl-L-arginine	2.72×10^4	4.41×10^4	insig
pmb2857	L-Glutamic acid-O-glycoside	7.03×10^3	3.78×10^3	insig
mws5035	L-Leucyl-L-phenylalanine	2.29×10^4	3.35×10^4	insig
pme0021	L-Phenylalanine	1.99×10^6	3.03×10^6	insig
pme0122	N6-Acetyl-L-lysine	6.18×10^4	1.02×10^5	insig
pme0026	L-Lysine	4.85×10^5	6.53×10^5	insig
Lmhp002031	L-Leucyl-L-Leucine	4.13×10^4	5.23×10^4	insig
pme2617	L-Methionine Sulfoxide	2.31×10^6	2.18×10^6	insig
pme1286	S-(5'-Adenosy)-L-homocysteine	2.17×10^4	2.59×10^4	insig
Zmgn001039	S-Ribosyl-L-homocysteine	1.16×10^4	1.61×10^4	insig
mws1587	L-Norleucine	6.16×10^6	8.57×10^6	insig
pme0193	L-Glutamine	4.66×10^5	5.97×10^5	insig
pmb0464	L-Aspartic acid-O-diglucoside	2.30×10^6	1.92×10^6	insig
mws0256	L-Valine	2.34×10^7	2.74×10^7	insig
pme3382	N-Acetyl-L-threonine	6.10×10^4	6.87×10^4	insig
pme0008	L-Citrulline	4.59×10^5	5.10×10^5	insig
pme1712	L-Saccharopine	4.62×10^3	6.28×10^3	insig
pme2758	4-Hydroxy-L-glutamic acid	2.02×10^4	2.45×10^4	insig
mws0260	L-Arginine	7.16×10^5	7.94×10^5	insig
pme0006	L-Proline	8.88×10^6	7.82×10^6	insig
pme0116	L-Carnosine	7.41×10^3	4.74×10^3	insig
pme1228	5-Hydroxy-L-tryptophan	5.86×10^4	5.58×10^4	insig
Lmbp000123	L-Homomethionine	3.01×10^4	2.63×10^4	insig
Zmgn002106	N-Acetyl-L-phenylalanine	2.86×10^5	3.29×10^5	insig
Lmqp000427	N-Methyl-Trans-4-Hydroxy-L-Proline	5.70×10^5	6.47×10^5	insig
pme1086	Glutathione reduced form	2.63×10^5	3.30×10^5	insig
pmb1283	L-Glutaminyl-L-valyl-L-valyl-L-cysteine	9.55×10^3	1.14×10^4	insig
pme3388	Homoarginine	7.89×10^3	9.67×10^3	insig
Lmhp002001	L-Valyl-L-Phenylalanine	2.80×10^4	3.23×10^4	insig
pme2559	N-Acetyl-L-Aspartic Acid	8.48×10^4	1.01×10^5	insig
pme0181	3-Methyl-L-Histidine	4.95×10^4	5.54×10^4	insig
mws0340	2,3-Dimethylsuccinic acid	9.51×10^3	8.09×10^3	insig
pme2735	S-Adenosyl-L-methionine	5.21×10^4	6.61×10^4	insig
mws0001	L-Asparagine	2.69×10^7	2.92×10^7	insig
mws0875	L-Cysteinyl-L-glycine	8.60×10^3	9.63×10^3	insig
mws4134	Oxiglutatione	2.29×10^4	2.56×10^4	insig
pme3193	N-Acetyl-L-glycine	4.92×10^3	4.35×10^3	insig

Rfmb320	1-Methylpiperidine-2-carboxylic acid	1.66×10^5	1.33×10^5	insig
pme0195	L-Cysteine	1.21×10^4	1.03×10^4	insig
Lmhp001670	L-Valyl-L-Leucine	4.29×10^4	4.78×10^4	insig
pme3827	3,4-Dihydroxy-L-phenylalanine (L-Dopa)	2.03×10^5	2.25×10^5	insig
mws0124	N-(3-Indolylacetyl)-L-alanine	2.63×10^4	2.51×10^4	insig
pme2122	Histamine	1.11×10^5	9.89×10^4	insig
mws0254	L-Histidine	7.06×10^5	9.30×10^5	insig
ML10181668	Cycloleucine	3.06×10^7	2.92×10^7	insig
mws0219	L-Aspartic Acid	5.34×10^6	5.10×10^6	insig
Lmrj002087	L-Isoleucyl-L-Aspartate	2.99×10^5	3.17×10^5	insig
pme0137	N-Acetyl-L-Glutamine	7.87×10^5	7.34×10^5	insig
Lmhp001430	Cyclo(Pro-Glu)	4.57×10^4	4.85×10^4	insig
mws0193	L-Homocitrulline	1.73×10^4	1.53×10^4	insig
mws5042	L-Glycyl-L-phenylalanine	2.08×10^4	1.94×10^4	insig
mws0629	L-Aspartyl-L-Phenylalanine	9.03×10^4	9.52×10^4	insig
pme3033	N,N-Dimethylglycine	1.46×10^5	1.51×10^5	insig
pme2566	5-L-Glutamyl-L-amino acid	1.70×10^4	1.64×10^4	insig
Zmzn000113	L-threo-3-Methylaspartate	7.43×10^5	7.29×10^5	insig
mws4176	L-Alanyl-L-Phenylalanine	6.88×10^3	7.03×10^3	insig
mws1050	O-Acetylserine	2.02×10^5	1.92×10^5	insig
pme3017	2-Aminoisobutyric acid	1.64×10^5	1.58×10^5	insig
mws0582	S-(Methyl)glutathione	1.09×10^6	1.02×10^6	insig
NK10251888	NG,NG-Dimethyl-L-arginine	6.27×10^4	6.69×10^4	insig
pme0170	N-Acetyl-L-Arginine	3.93×10^4	3.67×10^4	insig
pmb2855	L-Glutamine-O-glycoside	1.63×10^4	1.60×10^4	insig
pme2914	3-Hydroxy-3-methylpentane-1,5-dioic acid	3.44×10^5	3.47×10^5	insig
pme0278	2,6-Diaminooimelic acid	1.26×10^4	1.22×10^4	insig
Rfmb319	Pipecolic acid	1.09×10^7	1.08×10^7	insig

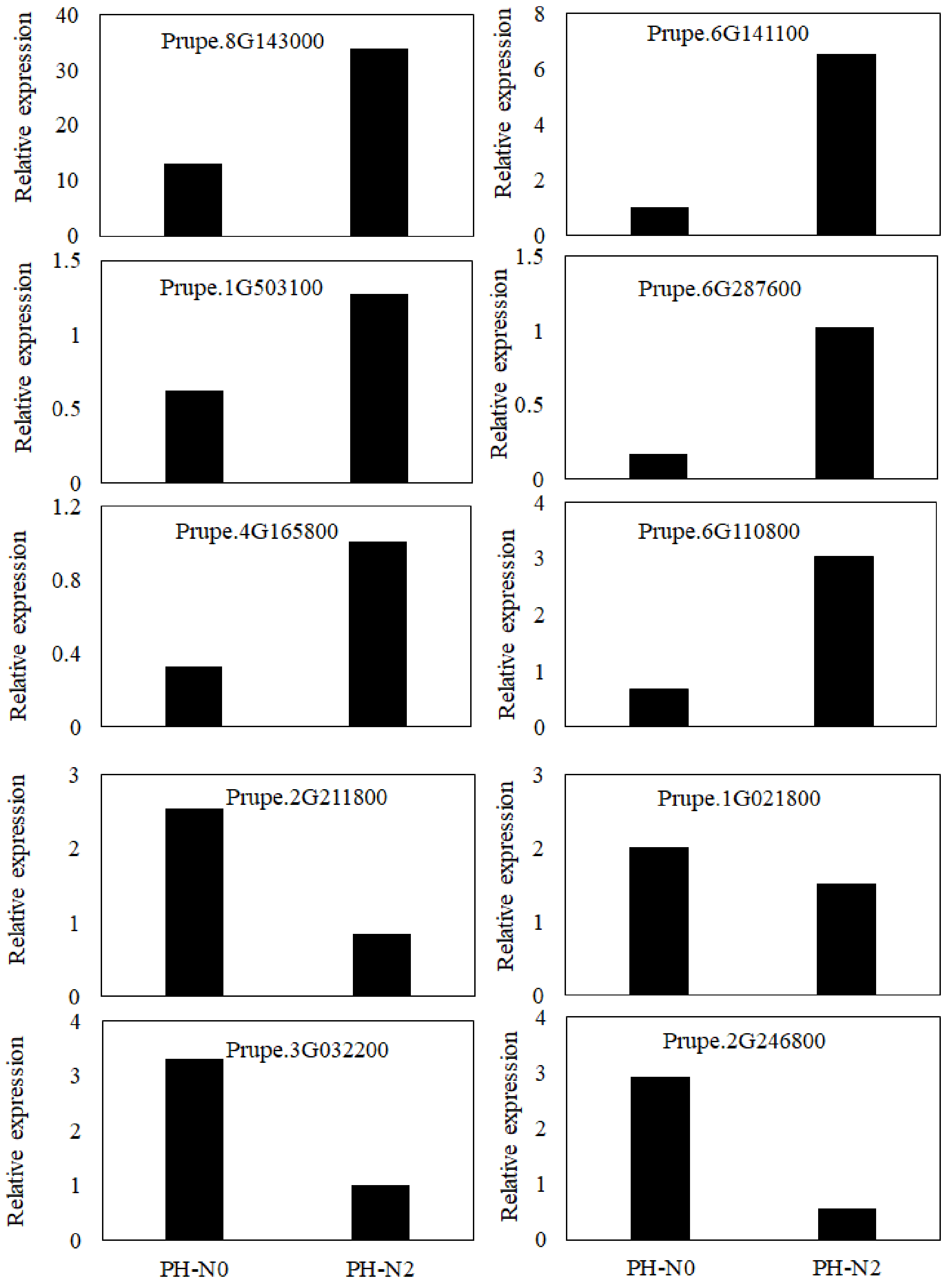

Figure S1. The relative expression of ten peach genes.
